# Ecology impacts the decrease of Spirochaetes and *Prevotella* in the fecal gut microbiota of urban humans

**DOI:** 10.1186/s12866-021-02337-5

**Published:** 2021-10-11

**Authors:** Louise B. Thingholm, Corinna Bang, Malte C. Rühlemann, Annika Starke, Florian Sicks, Verena Kaspari, Anabell Jandowsky, Kai Frölich, Gabriele Ismer, Andreas Bernhard, Claudia Bombis, Barbara Struve, Philipp Rausch, Andre Franke

**Affiliations:** 1 Institute of Clinical Molecular Biology, Christian-Albrechts-University of Kiel, Campus Kiel, Rosalind-Franklin-Str, 12, 24105 Kiel, Germany; 2 Tierpark Berlin-Friedrichsfelde GmbH, Berlin, Germany; 3 Tierparkvereinigung Neumuenster e.V, Neumuenster, Germany; 4 Tierpark Arche Warder e.V, Warder, Germany; 5 Tierpark Gettorf GmbH & Co. KG, Gettorf, Germany; 6 Zoo Leipzig GmbH, Leipzig, Germany; 7 Tierpark Hagenbeck Gemeinnützige Gesellschaft mbH, Hamburg, Germany; 8 Leintalzoo Schwaigern, Freudenmühle 1, 74193 Schwaigern, Germany; 9 Laboratory of Genomics and Molecular Biomedicine, Department of Biology, University of Copenhagen, Copenhagen, Denmark; 10 University Hospital Schleswig Holstein (UKSH), Campus Kiel, Kiel, Germany

**Keywords:** Mammals, Phylogeny, Ecology, Gut microbiota, Human health, Physiology

## Abstract

Compared to the huge microbial diversity in most mammals, human gut microbiomes have lost diversity while becoming specialized for animal-based diets – especially compared to chimps, their genetically closest ancestors. The lowered microbial diversity within the gut of westernized populations has also been associated with different kinds of chronic inflammatory diseases in humans. To further deepen our knowledge on phylogenetic and ecologic impacts on human health and fitness, we established the herein presented biobank as well as its comprehensive microbiota analysis. In total, 368 stool samples from 38 different animal species, including *Homo sapiens*, belonging to four diverse mammalian orders were collected at seven different locations and analyzed by 16S rRNA gene amplicon sequencing. Comprehensive data analysis was performed to (i) determine the overall impact of host phylogeny vs. diet, location, and ecology and to (ii) examine the general pattern of fecal bacterial diversity across captive mammals and humans.

By using a controlled study design with captive mammals we could verify that host phylogeny is the most dominant driver of mammalian gut microbiota composition. However, the effect of ecology appears to be able to overcome host phylogeny and should therefore be studied in more detail in future studies. Most importantly, our study could observe a remarkable decrease of Spirochaetes and *Prevotella* in westernized humans and platyrrhines, which is probably not only due to diet, but also to the social behavior and structure in these communities.

Our study highlights the importance of phylogenetic relationship and ecology within the evolution of mammalian fecal microbiota composition. Particularly, the observed decrease of Spirochaetes and *Prevotella* in westernized communities might be associated to lifestyle dependent rapid evolutionary changes, potentially involved in the establishment of dysbiotic microbiomes, which promote the etiology of chronic diseases.

**Supplementary Information:**

The online version contains supplementary material available at 10.1186/s12866-021-02337-5.

## Introduction

The fecal gut microbiota is well-recognized as an integral part of the mammalian gastrointestinal tract that is crucially involved in host physiology (reviewed in [1–3]). Particularly, the overall diversity of microorganisms and their provided functions play a key role in host nutrition, e.g., via energy conversion from otherwise indigestible foods [3, 4]. Further, the gut microbiota is central for the maturation and functionality of the mammalian immune response [5–7]. Consequently, changes in community composition have been shown to contribute to a variety of systemic diseases in humans, including inflammatory bowel disease (IBD), allergies, diabetes, and asthma [5, 8–10]. However, different environmental factors are known to substantially influence the gut microbiota, particularly host diet, but also the hosts’ evolutionary history [11]. With respect to this, it is assumed that microbial communities have influenced the evolution of multi-cellular organisms and to this end the evolution of humans [12]. Hence, one current research focus regarding microbial composition in health and disease is to understand the fundamental mechanisms shaping the gut microbiota of mammals. This knowledge can provide deep insights into the short- and long-term adaptation of bacterial communities to their respective hosts, as well as the potential role of maladaptation in disease pathogenesis.

Both diversity and function of the mammalian fecal microbiota have been characterized in several studies [11, 13–16]. Despite distinctions, the major bacterial phyla (Firmicutes and Bacteroidetes) and sometimes even the presence of specific genera have been shown to be highly conserved during the mammalian evolution [11, 12, 16, 17]. Additionally, key signature taxa compositions known as enterotype-like clusters have been found in various mammalian species [18–20]. Thus, in general, commonalities among mammalian species and their evolutionary history appears to be reflected in the composition and shared dynamics of their respective microbial communities. However, we currently lack understanding of the immense and recent changes in humans’ gut microbiota due to cultural diversification and urbanization and how this influences human health. Particularly in urban populations, a tremendous decrease of fecal bacterial diversity has been shown when compared to rural individuals; arguing for a strong and fast impact of lifestyle and diet [15, 21, 22]. With respect to this, it has been hypothesized that diet might mainly mediate the functional community assembly through environmental filtering, while host evolutionary history dictates the prevalence of specific heritable microbial taxa [11]. Nevertheless, the overall understanding of factors resulting in differences between species and individuals, such as genetics and environmental factors like geography, nutrition, and ecology is far from complete [11, 13–15, 23]. This might not only be due to technical artifacts throughout published studies (captive vs. wild animals, mammals vs. non-mammals, different laboratory workflows, distinct analytical approaches), but also the inability to disentangle the effect of correlated patterns such as the dietary behavior of animals, which largely follows their phylogeny. This dependence makes partitioning the microbial variation between the influences of diet and host phylogeny even more difficult and calls for diverse data sets and comprehensive analytical strategies considering intrinsic and extrinsic confounders.

In this study we analyzed 368 stool samples from 38 different mammal species including *Homo sapiens*, belonging to four diverse mammalian orders (Table 1). These samples included species from eight different zoos across Germany sampled over 4 years to analyze the effect of location and habitat as well as host phylogeny and ecology. All samples underwent the same technical procedure (DNA extraction and amplicon sequencing) to ensure compatibility as this has been a notable confounder in earlier studies [24]. Although the use of samples from captive animals is controversially discussed in the literature [3, 12, 22, 25], we here focused on studying the effects of host phylogeny and thus aimed to minimize confounding factors by the standardized food and rhythms in the diet of captive animals. The obtained microbial profiles were used to decipher similarities as well as differences in the fecal microbial composition between the various host species with a specific focus on the *Hominidae* family. Here, we also included a dataset comprising fecal bacterial microbiota results of both a children cohort from Guinea-Bissau, Western Africa, and an adult cohort from Democratic Republic Congo, Central Africa, and Ivory Coast, Western Africa, to study rapid lifestyle dependent changes, which underlines the loss of bacterial diversity in westernized populations and provide interesting new findings for future research [26–28].

**Table 1:** Summary of animals and samples included in the present study

**No. of Individuals**	**No. of Locations**	**Species**	**Species (scientific)**	**Characteristics**	**Genus**	**Family**	**Diet**	**Order**
6	2^a,b^	*zebu*	*Bos primigenius f. taurus*	Ruminants Social	Bos	Bovidae	Herbivores, feeding on grass, foliage, and plant products	Artiodactyla
7	3^a,b,c^	*goat*	*Capra aegagrus f. hircus*	Ruminants Social	Capra			
10	2^a,b^	*sheep*	*Ovis orientalis f. aries*	Ruminants Social	Ovis			
3	1^b^	*eland*	*Taurotragus oryx*	NonRuminant Solitary	Taurotragus			
2	1^b^	*camel*	*Camelus ferus f. bactrianus*	Ruminants Social	Camelus	Camelidae		
8	1^b^	*vicuna*	*Vicugna vicugna f. pacos*	NonRuminants Social	Vicugna			
2	1^c^	*elk*	*Alces alces alces*	NonRuminants Solitary	Alces	Cervidae		
5	1^c^	*reindeer*	*Rangifer tarandus fennicus*	Ruminants Social	Rangifer			
14	1^a^	*wild boar*	*Sus scrofa*	NonRuminants Social	Sus	Suidae	Omnivores, eating grass, leaves, roots, insects, worms	
23	2^b,d^	*ring-tailed coati*	*Nasua nasua*	NonRuminants Social	Nasua	Procyonidae	Omnivore	Carnivora
21	2^c,d^	*racoon*	*Procyon lotor*	NonRuminants Solitary	Procyon			
2	1^c^	*black bear*	*Ursus americanus*	NonRuminants Solitary	Ursus	Ursidae		
4	2^c,d^	*polar bear*	*Ursus maritimus*	NonRuminants Solitary			Carnivore	
2	1^b^	*meerkat*	*Suricata suricatta*	NonRuminants Social	Suricata	Herpestidae		
13	1^a^	*donkey*	*Equus asinus*	Ruminants Social	Equus	Equidae	Herbivores, feed on grasses, leaves, and other plant parts (hindgut fermenters)	Perissodactyla
11	3^a,b,c^	*horse*	*Equus ferus caballus*	Ruminants Social				
4	1^b^	*zebra*	*Equus quagga boehmi*	NonRuminants Social				
2	1^b^	*tapir*	*Tapirus terrestris*	NonRuminants Solitary	Tapirus	Tapiridae		
19	1^d^	*marmoset*	*Callithrix jacchu*	NonRuminants Social	Callithrix	Callitrichidae	Omnivores, eating insects, fruit, and the sap or gum from trees	Primates
1	1^b^	*emperor tamarin*	*Saguinus imperator subgrisescens*	NonRuminants Social	Saguinus			
3	1^b^	*white-lipped tamarin*	*Saguinus labiatus*	NonRuminants Social				
2	1^c^	*red-handed tamarin*	*Saguinus midas*	NonRuminants Social				
3	1^b^	*cotton-top tamarin*	*Saguinus oedipus*	NonRuminants Social				
5	1^b^	*squirrel monkey*	*Saimiri sciureus*	NonRuminants Social	Saimiri	Cebidae		
2	1^b^	*Diana monkey*	*Cercopithecus diana*	NonRuminants Social	Cercopithecus	Cercopithecidae	Omnivores, eating mainly fruits, but also flowers, leaves, bulbs and rhizomes, insects, snails, small mammals	
1	1^b^	*black crested mangabey*	*Lophocebus aterrimus*	NonRuminants Social	Lophocebus			
6	1^b^	*celebes crested macaque*	*Macaca nigra*	NonRuminants Social	Macaca			
37	3^b,c,d^	*barbary macaque*	*Macaca sylvanus*	NonRuminants Social				
2	1^e^	*drill*	*Mandrillus leucophaeus*	NonRuminants Social	Mandrillus			
1	1^f^	*mandrill*	*Mandrillus sphinx*	NonRuminants Social				
3	1^e^	*hamadryas baboon*	*Papio hamadryas*	NonRuminants Social	Papio			
48	2^g, b^	*human*	*Homo sapiens*	NonRuminants Social	Homo	Hominidae	Omnivores, with fruit as the preferred food among all but some human groups	
1	1^h^	*bonobo*	*Pan paniscus*	NonRuminants Social	Pan			
56	2^b, f^	*chimp*	*Pan troglodytes*	NonRuminants Social				
6	1^e^	*Sumatran orangutan*	*Pongo abelii*	NonRuminants Solitary	Pongo			
12	2^b, f^	*white-handed gibbon*	*Hylobates lar*	NonRuminants Social	Hylobates	Hylobatidae	Omnivores, eating mainly fruits, but also flowers, leaves and insects	
10	2^b, c^	*ring-tailed lemur*	*Lemur catta*	NonRuminants Social	Lemur	Lemuridae	Omnivores	
11	2^b, c^	*red ruffed lemur*	*Varecia rubra*	NonRuminants Social	Varecia		Herbivorous, eating mainly fruits and leaves	

A total of 38 different mammalian species were sampled across seven different locations in Germany. The table summarizes the number of animals per species, the locations where each species was sampled, the phylogeny of the species, their characteristic and dietary behavior

^a^Arche Warder, ^b^Gettorf, ^c^Berlin, ^d^Neumuenster, ^e^Hagenbeck, ^f^Schwaigern, ^g^Kiel, ^h^Leipzig

## Results

### Study cohort

In total, 426 stool samples from 42 different animal species including *Homo sapiens*, belonging to four diverse mammalian orders were collected and microbiome profiled. After filtering (see Materials and methods) the dataset included microbiota profiles from 368 unique subjects across 38 mammalian species, including four zookeepers, 44 non-zoo-keeping humans and 320 zoo animals (Table 1). These animals were sampled in seven different zoos across Germany (Berlin, Neumuenster, Gettorf, Warder, Hamburg, Leipzig, Schwaigern) to analyze the effect of location and habitat as well as host phylogeny and ecology. Mammalian orders in the cohort comprise Artiodactyla, Carnivora, Perissodactyla and Primates. Artiodactyla (also called even-toed ungulates) encompass most of the world’s species of large land mammals such as sheep, goats, camels, pigs, cows, and deer, from which nine species are included in this study. Four different species were included from the order Perissodactyla (also called odd-toed ungulates), which in general consists of about 17 species that are hoofed animals (e.g.*,* horses and rhinoceroses). The order Carnivora comprises over 280 species of placental mammals, from which five were sampled in this study. Additionally, 20 different species from the order Primates were analyzed, including the most closely human related primate species, *Pan paniscus* and *Pan troglodytes*.

### Fecal microbiota composition highly reflects animals’ phylogeny and thus, also their diet

In this study, the V1V2 region of the 16S rRNA gene from fecal samples were sequenced and ASV tables were generated and annotated to species level. This resulted in 63,780 unique ASVs and 1381 species across the 426 samples (agglomerated keeping species-level unannotated ASVs). Subject and sample filtering, including the selection of one sample per animal (or stool pool) resulted in 368 samples from 38 species (see Materials and methods). A pattern of specificity of microbial species to subgroups of hosts was apparent since 80.5% of microbial species were detected in less than 5% of the samples, likely reflecting the high diversity and distinctness of the various host species and their respective ecologies in the dataset [11].

**Fig. 1:**
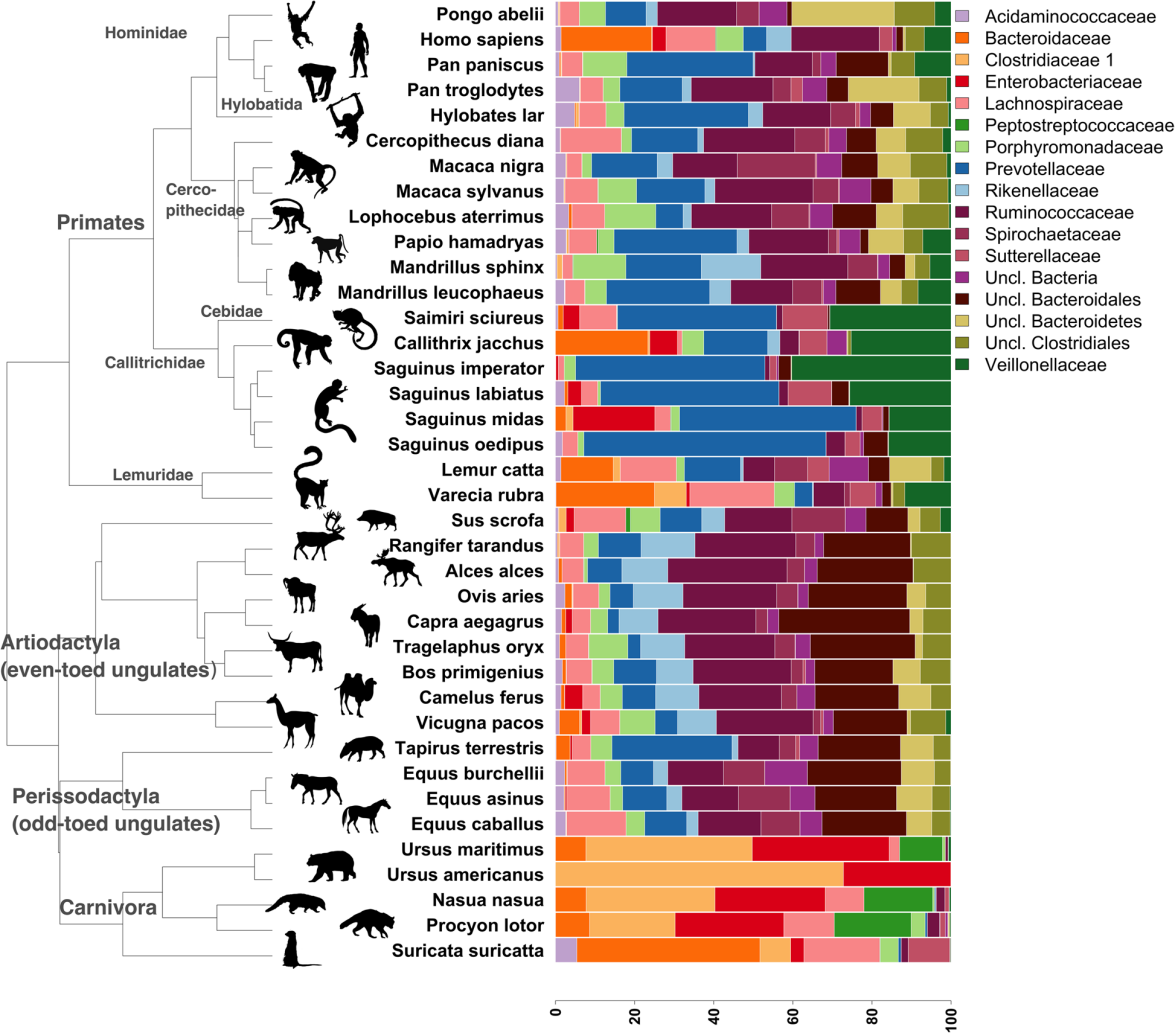
Mammals gut bacterial profile by host phylogeny. Mapping of family-level microbiome mean relative abundances (17 most abundant) onto host phylogenetic tree (built using http://timetree.org/) revealed clear clustering of microbiome profile by host clade. A total of 38 different host species with microbiome data are included, and microbiome data aggregated at family level keeping unannotated clades (seen as uncl. (unclassified) in legend). Icons taken from http://phylopic.org/. Credits to Rebecca Groom, Roberto Díaz Sibaja, Sarah Werning

As expected and observed earlier (recently reviewed in [29]), the fecal microbiota profiles of the studied mammalian species followed their phylogenetic relationships regarding prevalence and abundance of microbial families (Robinson-Foulds distance between the host phylogenetic tree and the microbial dendrogram (Bray-Curtis and Jaccard), median *p* = 0.001; Mantel comparison of distance matrices from the host phylogenetic tree and species abundance table, median *p* = 0.001; both methods with Bray-Curtis and Jaccard diversity measures and permutations as described in methods. Fig. 1). The stacked bar plot in Fig. 1 illustrates the average microbiota profile of each selected host species in the study and highlights how substantially the Carnivora microbiota differ from the remaining host clades. Another interesting finding from this evaluation was the restricted abundance of *Bacteroidaceae*, which only showed mean relative abundances above 10 in *Homo sapiens*, *Callithrix jacchus*, *Varecia rubra* and *Suricata suricatta*. Relatively high proportions of unclassified bacterial families were found in the orders Artiodactyla and Perissodactyla, underlining the understudied fecal microbial diversity within these orders. Evaluating the prevalence of species in each of the four mammalian orders, we found support for Carnivora as the limiting group to define a mammalian core microbiota. Out of 603 microbial species, five ASVs were found with a prevalence above 50% in all orders, one belonging to each of *Bacteroidaceae*, *Ruminococcaceae, Erysipelotrichaceae, Lachnospiraceae* and unclassified *Candidatus Saccharibacteria*, however none of them were annotated at species level. When disregarding the Carnivora clade, 27 species met the threshold. When Artiodactyla, Perissodactyla or Primates in turn were left out, only 5, 7 or 6 species met the threshold, respectively, highlighting the high degree of community differentiation between the groups.

To evaluate if microbial species showed specificity to any single or combination of host clades, we applied a multi-level pattern analysis (indicspecies::multipatt). We found 305 species with significant specificity (out of 602 tested) to any one combination of host order clades, with 82 assigned to Artiodactyla, 156 to Carnivora, 114 to Perissodactyla and 83 to Primates (multipatt, p.adj < 0.05, Table S1). Interestingly, a large percentage of species associated with Carnivora were uniquely associated with this clade (139 of 156) and therefore are possible indicator species for this order and include two species of *Escherichia/Shigella* genus, and 21 species of the order Clostridiales. This pattern was supported by a complimentary indicator-species analysis which identified 124 indicator species for Carnivora (indicspecies::indicators, p.adj < 0.05), including the two *Escherichia/Shigella* clades. The indicator-species analysis (indicspecies::indicators) found 54 indicator species for primates (p.adj < 0.05), thereby supporting the pattern observed in the multipatt-analysis, that primates have fewer indicator species and therefore a less unique microbiome, as compared to the Carnivora.

We then evaluated the phylogenetic relatedness of individual microbial species to see if their relative abundance is associated with host phylogeny and found a multitude of microbial associations with host phylogeny. We calculated the mean phylogenetic distance (MPD) between all microbial species and compared the observed pattern to that expected under a random community composition obtained by randomization while holding species richness constant, as we observed some association of host phylogeny with microbiome alpha diversity (picante::ses.mpd). We selected microbial species present in > 5 samples and regressed out the effect of location (see Materials and methods). A total of 231 microbial species (out of 536) showed phylogenetic relatedness (p.adj < 0.05, abundance weighted MPD model, 999 permutations, Table S2). Two *Prevotella*, namely *Prevotella copri* and one unclassified at species level, were tested in the model and both showed significant phylogenetic relatedness (p.adj < 0.05). Five species of Spirochaetes were analyzed, with three Spirochaetales showing significant phylogenetic relatedness (all unclassified at species level, two annotated as *Sphaerochaeta* and *Treponema* at genus level). The remaining two Spirochaetes showed nominal association but did not pass multiple testing correction (*p* = 0.026 and 0.047).

Dietary preferences are one factor that strongly varies between mammalian orders and diet is believed to be one of the most important factors influencing the gut microbiome, in addition to host genetics, ecology, or habitat. However, as (i) host-phylogenetic clusters have strongly correlated dietary behaviors which largely follow host phylogeny, (ii) our dietary data is restricted to main food categories as binary data e.g. overall intake of meat and/or plant-based diet, and (iii) as dietary data is only available for approximately one third of the cohort (incl. only two Carnivora), consistent and reliable segregation of the microbial variation between diet and host phylogeny is not fully feasible using this dataset (strongly nested). Still, with a large overlap in diets for different species and some within-species differences due to between-zoo differences, we found it of relevance to consider the dietary patterns as much as the data allowed.

First, we evaluated if dietary information could explain parts of the variation in the gut microbiota community composition of the mammals. To this end, we used the multiple regression on matrices (MRMs) model, as described in more detail below. Details on diet were available for 115 hosts belonging to 15 species. The data was used to generate eight dietary variables that reflect the main dietary categories from the animal diets e.g. fruit, meat, vegetables, greenery (see Materials and methods), and a distance matrix was calculated based on shared dietary patterns (Jaccard distance). We calculated the variation explained by phylogeny, location and diet using the 115 samples with available dietary data. In this subset of samples, both with and without the inclusion of dietary data, the effect of host phylogeny was significant (median *p* < 0.05). Diet was not significant (median *p* > 0.05) despite 12% (median) variation explained, probably due to the high correlation between diet and host phylogeny in general. Above we observed an association of *Bacteroidaceae* with host phylogeny and identified two *Prevotella* species showing phylogenetic association with the host (namely *Prevotella copri* and *Prevotella sp*.). Abundance patterns of *Prevotella* has previously been found to be associated with diet with positive associations with fiber intake and negative associations with meat intake. Therefore, we zoomed in on these two species and evaluated the role of vegetables and meat on their abundance while considering location and host phylogeny via Phylogenetic Generalized Least Squares models (PGLS). The unclassified *Prevotella* significantly associated with meat intake (and host phylogeny and location) (PGLS, meat *p* < 0.05, lambda = 0.34 (95% CI 0.16-0.61)), while *Prevotella copri* only associated significantly with host phylogeny (lambda = 0.96 (95% CI 0.90-0.98)). At the family level, we evaluated association of *Bacteroidaceae* with meat intake and identified both meat and host phylogeny as significantly factors influencing its abundance across hosts (PGLS p < 0.05, lambda = 0.49 (95% CI 0.26-0.74)).

**Fig. 2:**
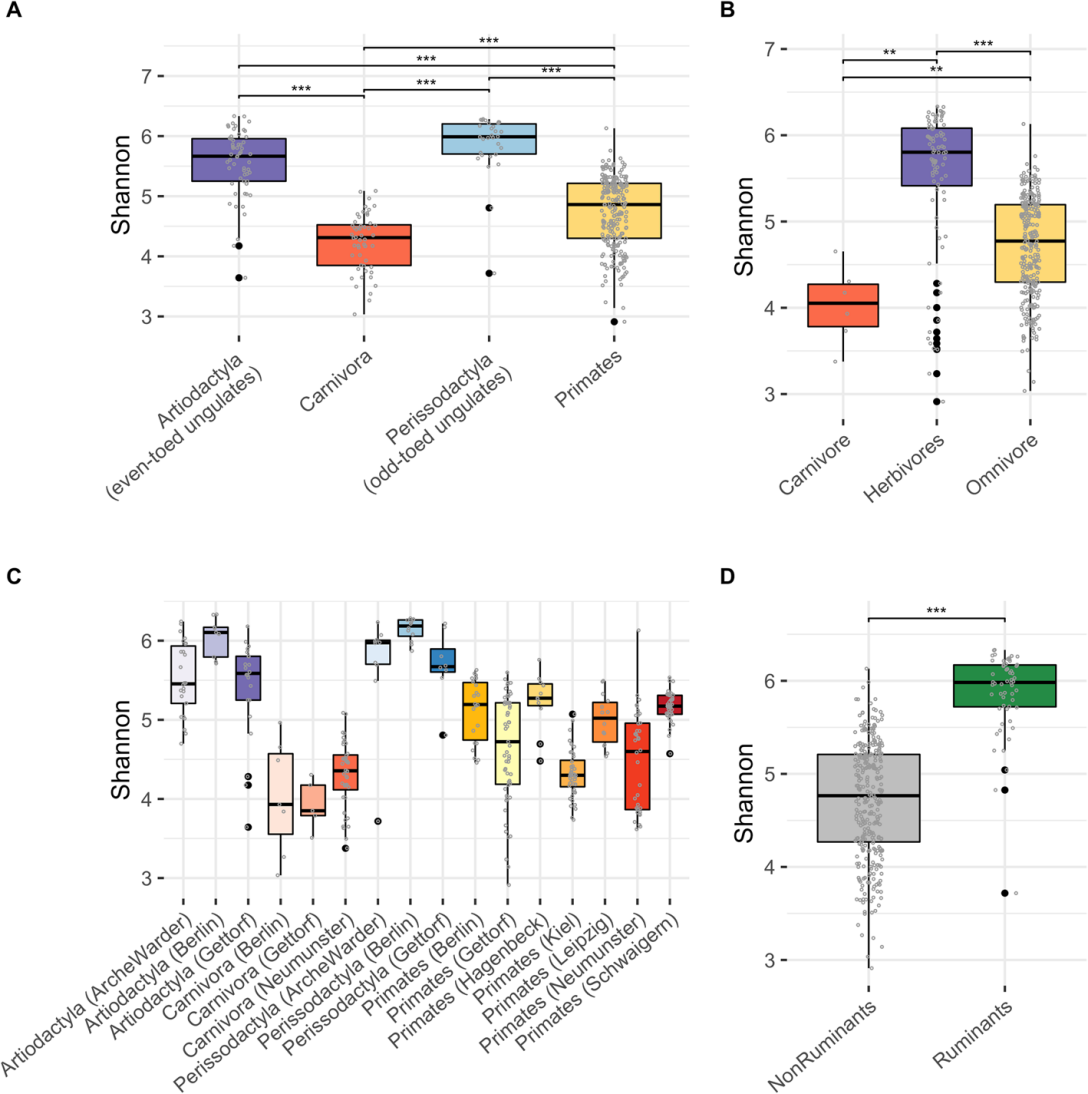
Comparison of alpha diversity between host clades. **A** At order-level, **B** by dietary behavior, **C** order level sub-stratified by sampling location and (D) ruminant phenotype. Alpha diversity measured as Shannon diversity, differed between host order clades in a manner that largely reflected dietary preferences but with little association to location. Analysis of pairwise differences was performed using ANOVA, correcting for location. ***: p.adj < 0.001, **: p.adj < 0.01

To further understand the influence of dietary preferences on microbiome diversity, we compared the alpha diversity between hosts grouped into their four orders. Shannon diversity varied between all pairs of host orders that were not both predominantly herbivores (two-way ANOVA correcting for location, q < 0.001), while pairs comprising predominantly herbivores showed no significant difference (q > 0.05) (no difference between Perissodactyla and Artiodactyla) (Fig. 2). Carnivora (carnivores and omnivores) and Primates (predominantly omnivores) had on average approximately half the community diversity of herbivores (Artiodactyla and Perissodactyla), probably highlighting the diversity increasing effect of higher plant and fiber intake, which requires a rich enzyme repertoire. This observation of a diet-driven alpha-diversity pattern was further supported by a comparison of Shannon diversity between mammals grouped by their dietary behaviour (as opposed to taxonomic order) (Fig. 2B). The observation of a pattern of alpha diversity that follows both the dietary behaviour and host phylogeny of the mammals, was further supported by a Phylogenetic Generalized Least Squares (PGLS) analysis of the association between alpha diversity and dietary behaviour, that becomes insignificant when considering host phylogeny (pgls in R package caper, lambda = ‘ML’, controlling for location, *p* > 0.05 for both Shannon and Chao). Interestingly, alpha diversity varied greatly among the Primates. At the genus level, *Hylobates*, *Macaca*, *Pan* and *Pongo*, showed the highest diversity, while *Varecia* and *Callithrix* showed the lowest diversity (considering clades with min. 5 individuals, Figure S1). A similar pattern was found for richness (Chao), with the lowest diversity found in Carnivora and Primates (Figures S1 and S2). For richness, Perissodactyla showed a high diversity also compared to the other clade of predominantly herbivores (versus Artiodactyla q = 6.2 × 10^− 5^). Host phylogeny directly dictates the animal’s dietary preferences in part by shaping their digestive abilities such as the ruminant animals specialized stomach that give them the ability to acquire nutrients from plant-based food. The fermenting process is driven by microbial actions, and when comparing the microbial diversity between ruminants and non-ruminants in the dataset, we identified a significantly higher diversity in the ruminant mammals (Fig. 2D). To evaluate if there was a detectable effect of individual food groups when controlling for phylogenetic relatedness and location, we applied a PGLS model to the 115 samples with available dietary data and evaluated the association of each of the eight food groups with Shannon diversity. The analysis detected a significant association for fruit, eggs and greenery (PGLS, *p* < 0.05) and a trending association for multimineral/vitamin (*p* = 0.05), all models retaining a significant lambda, indicating a role of both host phylogeny and intake of these food groups on microbial diversity.

To evaluate the variability of microbial communities within host groups with different dietary preferences (carnivores *n* = 6, herbivores *n* = 84, omnivores *n* = 278), we performed dispersion analysis based on the Bray-Curtis diversity measure of dissimilarity between host’s microbiome compositions (betadisper in R, bias.adjust = T). The analysis was performed for microbial taxa at the ASV, species, genus and family level, and at all levels the analysis detected a significant difference in variability. However, the pattern of variability between the diet groups changed when moving from the ASV to the higher taxonomic levels. At ASV level, the carnivores had the lowest dispersion, while there was no significant difference between the herbivores and omnivores (mean distance 0.59, 0.67, and 0.68, respectively). At species level, the herbivores diversity decreased drastically (mean distance 0.35), while the omnivores also decreased to 0.52 and carnivores remained largely unchanged (0.55). In addition, there was a significant difference between the herbivores and omnivores at species level (median *p* = 0.0017). The carnivores changed from having the lowest dispersion to having the highest, just above the omnivores, and the pattern remained stable at higher microbial taxonomic levels. Even as we adjusted for sample bias, notable variation in the number of different species sampled within each host order clade remained. When calculating the difference between the herbivores and omnivores, we therefore performed 100 random samplings of five host species groups per diet-group, performed the analysis of variation on each subsampling and then calculated the mean dispersion and *p*-value across the 100 analyses. The observed pattern is likely caused by the lower species assignment rates in the plant-eating groups as compared to carnivores; carnivores had the lowest number of ASVs with 328 ASVs, and omnivores the highest with 6198 ASVs. At microbial species level the herbivores had 396 species (incl. unannotated) down from 3006 ASVs. The relative change in richness was very low for carnivores (1.77 times) as compared to herbivores and omnivores (7.59 and 10.58, respectively). The carnivores showed the highest percentage of annotated ASVs across microbial species, genera and family levels, followed by the omnivores (percentage annotated microbial families: 99.4% for Carnivora, 75.7% for Omnivore and 67.0% for Herbivore).

### Host phylogeny remains an important factor in shaping gut microbiome also for captive and geographically separated mammals

Next, we evaluated whether the variation in the gut microbiota of the mammals is mainly explained by location (given by the Zoo’s geographical locations and humans continent of residence) or by their phylogenetic relatedness. We included one sample per individual mammal (as some mammals had been sampled multiple times) and used multiple regression on matrices (MRMs), as described previously [11], to calculate how much of the microbial variation could be assigned to host phylogeny and location. The three distance matrices were based on geographic coordinates for location, patristic distances for host phylogeny and the species table for the microbiota (see Materials and methods). To control for the effects of intra-species variation, we performed the analysis 100 times, each time with one randomly selected sample per host species. Thirty-eight host species had data points across all three matrices, and data was selected from a total of 368 samples. The analysis was performed considering both relative abundances (Bray-Curtis, BC) and presence/absence (Jaccard) for microbiota composition. In both analyses, host phylogeny explained a significant amount of variation (median *p*-value< 0.05, coefficient ~ 23% for BC and Jaccard), while the variation explained by location was insignificant (median *p*-value> 0.05, coefficient − 0.03% BC, − 0.09% Jaccard, Figure S3). In contrast, alpha diversity showed strong associations to the geographic location and the phylogenetic relationships between the animals (Shannon lambda = 0.77 (95% CI 0.63-0.87, lower and upper *p* < 0.05), location p < 0.05, *R*^2^ = 10%; Chao lambda = 0.80 (95% CI 0.68-0.89, lower and upper p < 0.05), location p < 0.05, *R*2 = 17%).

**Fig. 3:**
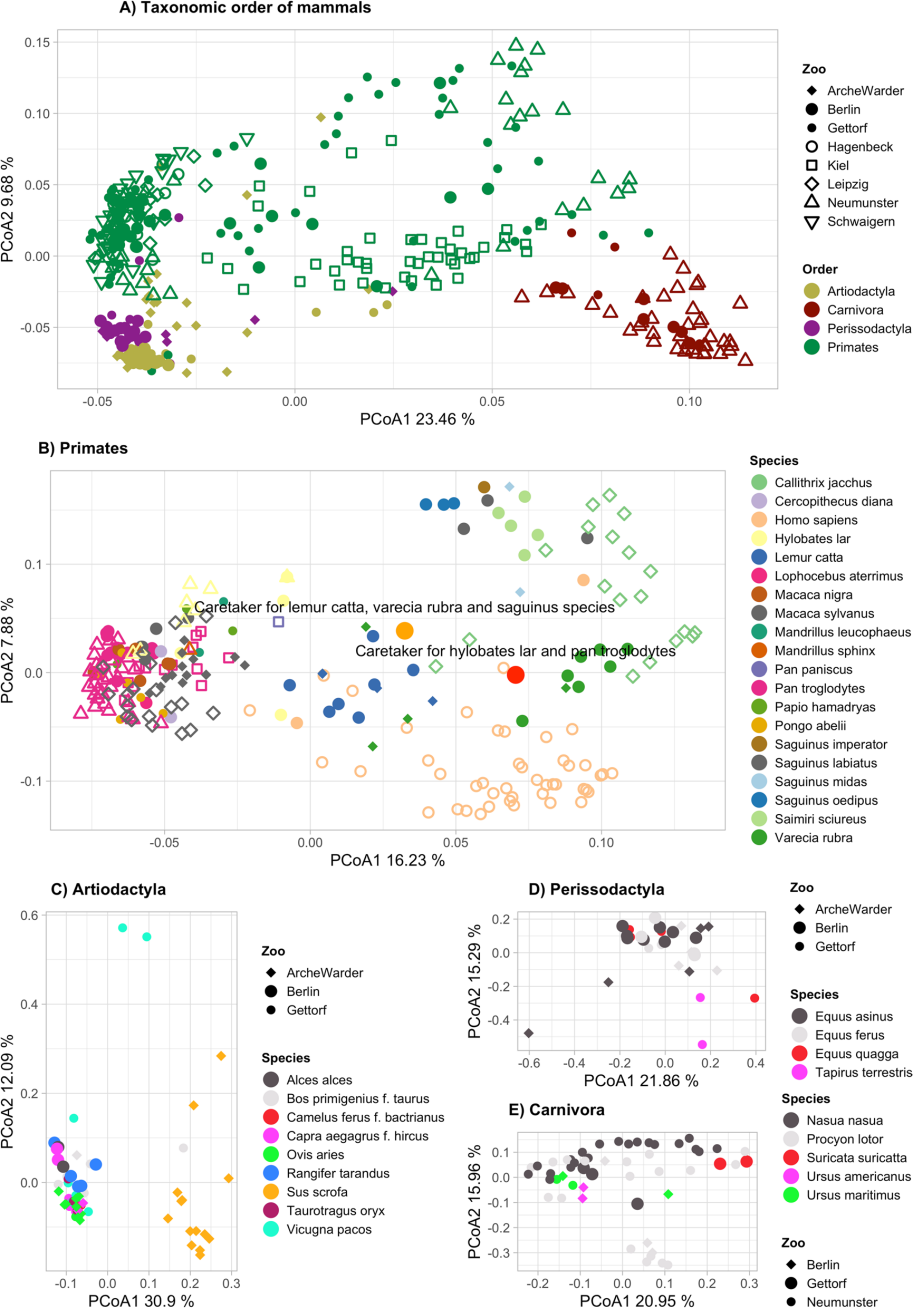
Graphical summary of the community structure by host phylogeny and zoo location. Panel **A** show host animals colored by phylogenetic order and shaped by location (Zoo or hometown) indicating only small effects of location. Panels **B**-**E** show animals belonging to each of the four different phylogenetic orders samples in the cohort. Panel **B** include enhanced circles and labels for the two animal zookeepers included in the dataset (shapes same as for A). The clustering of species in those orders highly reflects their phylogeny, though many of them live in different group sizes and with diverse other species within the zoos itself. Each panel shows host animals colored by phylogenetic species and shaped by location (Zoo or hometown). Plots are unconstrained principal coordinates analyses made with vegan::capscale, with dist = “bray”, metaMDS = F. Percentages given at each axis present the proportion of variance explained on the axis

Despite the very limited variation in microbial community composition found to be explained by location when using the full host phylogenetic tree reflecting geological time and location reflecting geographic distance, further evaluation of the host phylogenetic subgroups using ANOVA and Permutational MANOVA (adonis) approaches did detect some influence of location. These analyses treat location and host phylogeny as categories unorganized by evolutionary distance or morphology (host taxonomy), or geographical distance (here zoo location). For host mammals grouped at genus level, the variation in microbial composition explained by location was 3.36% after adjusting for phylogeny, and variation explained by phylogeny was 34.7% (likewise after adjusting for location). These associations were highly significant (adonis2 *p* < 0.001, species-level microbiome, 999 perm, min. 5 animals per host group, Table S3). Visual evaluation of the community structure by host phylogeny and zoo location supported an effect of both factors (Fig. 3), however only a small shift could be detected due to location. Whereas the order Carnivora again clustered decidedly different from all others, members of the Artiodactyla and Perissodactyla were more similar to each other even compared to the Primates, which displayed high variation (dispersion) among their microbial communities. Having a closer look into each order, Primates revealed a peculiar pattern in their microbial communities. Here, four human samples were included that did not belong to the human Kiel control group, but instead were sampled from two animal zookeepers from Gettorf, as well as from two workers not handling animals. The two samples of the animal zookeepers shifted, away from the human samples from the geographically close Kiel area, towards Gettorf zoo where they worked and for the zookeeper of lemurs, tamarins and squirrel monkeys, the shift was directed towards the *Saguinus oedipus* (tamarins) (see Fig. 3 “Primates”), indicating their microbiomes are influenced by the animals they interact with and location. Otherwise, the clustering of primate species highly reflects their phylogeny, even though many of them live in different group sizes and together with many other species. A similar pattern could be observed for most other host orders, too, including sheep and goats within Artiodactyla or racoons within Carnivora.

When considering the within-sample diversity (alpha diversity) using PGLS analysis, as opposed to the community composition (beta diversity) evaluated above, support was detected for an effect of both host phylogeny and location, supporting the above observations for community structure (Shannon lambda = 0.77 (95% CI 0.63-0.87, lower and upper *p* < 0.05), location *p* < 0.05, *R*^2^ = 10%; Chao lambda = 0.80 (95% CI 0.68-0.89, lower and upper *p* < 0.05), location *p* < 0.05, *R*^2^ = 17%).

One way by which the confinement of animals to specific zoos could influence a possible phylogeny-driven microbial composition could be through local community dynamics and restricted bacterial/host dispersal between locations, eventually leading to zoo specific microbial communities/signatures. Thus, we looked for zoo-specific microbial species within the 351 most abundant microbial species (min abundance 0.001% in at least 3% of samples). Only four species (*Alistipes finegoldii*, *Bacteroides stercoris*, *Bifidobacterium tissieri* and *Clostridium leptum*) were unique to one location, namely *A. finegoldii* and *C. leptum* to Kiel, and *B. stercoris* and *B. tissieri* to Neumuenster. The bacteria specific to Kiel are present only in human hosts while bacteria specific to Neumuenster Zoo are present in Primates and Carnivora. In Neumuenster, marmosets (Primates) hosted by far the majority of the two species (found in 17 or the 19 marmosets’ stool-pool samples), while samples from marmosets were also only available for this location. Both *B. stercoris* and *B. tissieri* were also found in one stool-pool from ring-tailed coati, and *B. tissieri* was found in one stool-pool from racoons. Both ring-tailed coatis and racoons were also sampled in other zoos (Gettorf and Berlin, respectively), indicating some cross-host species transfer within zoos or similarities in the host’s ecologies. However, the overall pattern does not indicate widespread zoo-specific microbial species. A PGLS analysis of *B. stercoris* and *B. tissieri* with location, confirmed the importance of host phylogeny over location (caper::PGLS, lambda = ‘ML’, *B. stercoris* lambda = 0.75 (95% CI 0.61-0.85, p upper and lower < 0.05), location *p* = 0.85; *B. tissieri* lambda = 0.67 (95% CI 0.50-0.80, p upper and lower < 0.05), location *p* = 0.74). PGLS cannot be used to evaluate the two taxa unique to Kiel due to Kiel location only containing samples from one host species (44 of 48 German humans).

### Variation in the family Hominidae

A reduced diversity and an increase in dispersion have been observed for humans as compared to closely related taxa or other mammals. Across mammalian families, Hominidae showed a highly variable diversity (Figure S1). The range overlapped with most other families but was clearly lower than in most herbivorous families and showed generally higher Shannon diversity than the Carnivora, and the primate families Callitrichidae (marmoset), Cebidae and Lemuridae. Our dataset included three genera, *Homo, Pan* and *Pongo* (comprised of four species), within the family *Hominidae*. Since microbial diversity is also known to be decreased within westernized populations [15, 21], we here included two additional cohorts with fecal bacterial microbiota data; one dataset comprised of a large children cohort from urban Bissau, the capital of one of the poorest countries in the world, Guinea-Bissau (Western Africa) [26, 30]. We selected 159 individuals who were recruited at home (controls) at a minimum of 10 years of age. While the gut microbiome of children above the age of five is generally found to be adult-like, we included a second cohort of 24 adults, 12 from Ivory Coast and 12 from DR Congo. This reduces any potential age-bias and increases the geographic diversity of the non-westernized human subgroup. We compared the alpha diversity of the non-westernized human subjects with the German (Kiel) human subjects of our study cohort. With regard to Shannon diversity, African subjects had a significantly higher diversity compared to the German subjects (*p* = 9.2 × 10^− 12^). There was no significant difference between the diversity of the African adult and child’s subgroups (q = 0.38), and both had lower diversity when compared to the hominid primate genera *Pan* (q = 1.7 × 10^− 8^ and q = 2.2*10^− 16^). The difference to *Pongo* was not significant likely reflecting the limited analytic power for this genus (q = 0.13 and q = 0.057) (Figure S4). This placed the alpha diversity of the African humans in-between the diversity levels of the German humans and non-human representatives of the Hominidae family. PGLS analysis confirmed the importance of phylogeny in shaping diversity of these four hominid species clades (PGLS with location, lambda = ‘ML’, including only German humans, Shannon lambda = 0.73 (95% CI 0.33-0.95, *p* < 0.05), Chao lambda = 0.83 (95% CI 0.51-0.97, p < 0.05). Finally, in addition to the diversity differences between microbial communities in the Hominidae, we could identify a higher community variability in humans compared to apes (ANOVA on betadisper object, *p* < 0.001), particularly in German (Kiel) subjects (median distance to centroid; DR. Congo 0.34, Guinea-Bissau = 0.33, Ivory Coast = 0.36, Kiel = 0.42; ANOVA *p* = 0.002 for Kiel vs joined Africa subjects).

**Fig. 4:**
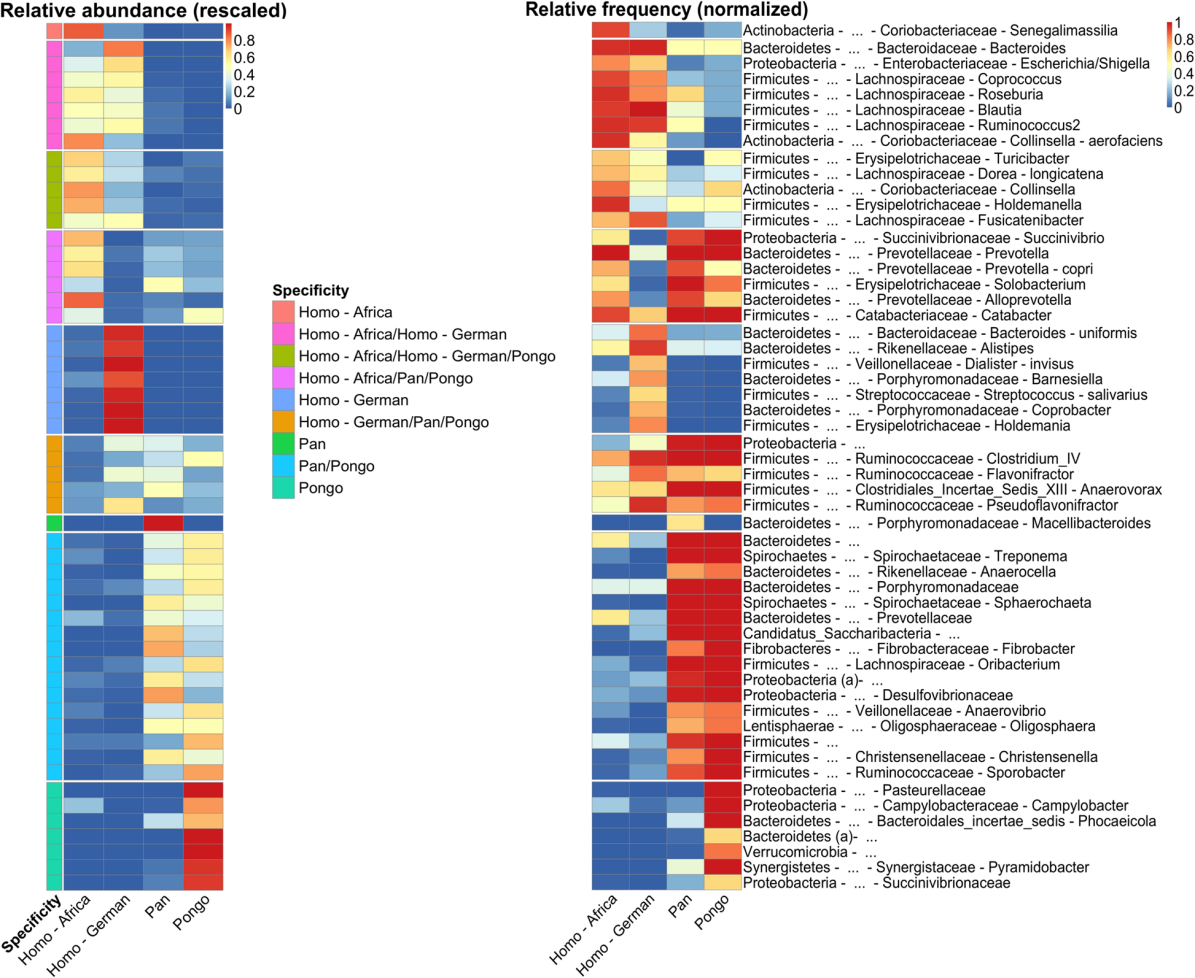
Microbial variation within the Hominidae family*.* Heatmap of Hominidae displaying the relative abundance (rescaled by rowsum for each species) and frequency of the 55 most significant species found by multi-level pattern analysis (multipatt in R package indicspecies), which was used to identify species that showed specificity in terms of abundance and prevalence to one of the subgroups *Pan*, *Pongo*, German or Guinea-Bissau human subjects, or a combination of those. Figure generated using pheatmap::pheatmap and arranged using Inkscape [26]. Relative abundance is rescaled. ASVs annotations at phylum, family, genus and species level are included, and a letter (a or b) distinguish ASVs annotated to same taxonomic level

To further explore the community differences in the Hominidae, we used the indicator species analysis (multipatt introduced above) to identify bacterial species that are specific to a certain host group (*Pan*, *Pongo*, Germans and Africans including both children and adult individuals). The analysis identified 145 species (out of 351 analyzed) with significant specificity to one or a combination of groups, with 2 assigned specifically to humans from Africa (out of 41 assigned to a combination that include Africans), 36 specifically to humans from Germany (out of 77), 8 specifically to *Pan* (out of 61) and 19 specifically to *Pongo* (out of 75) (multipatt p.adj < 0.05). Figure 4 shows the relative abundance and frequency of the 55 most significant species (p.adj < 0.001), the full list is presented in Table S4. Of the 36 species showing specificity to the German humans, eight belonged to the *Bacteroides* clade and six to *Alistipes*. Prior studies comparing mammal clades and westernized with non-westernized populations reported that Spirochaetes are increasingly absent from populations consuming a westernized diet [14, 21]. The current dataset includes six species assigned to the order Spirochaetes, of which four showed significant association to non-human subgroups, while two were highly abundant and prevalent in humans from Africa (*Brachyspira pilosicoli* and *B. aalborgi*). Also, *Prevotella* showed an association towards non-westernized subjects (*Prevotella copri* and one unclassified *Prevotella*, Table S4). Contrary to *Prevotella*, *Bacteroides* displayed a high abundance and prevalence only in human subjects from Germany (specificity to German humans, eight of nine analyzed *Bacteroides*, Table S4). These findings outline previously reported and yet unknown phenomena along gradients of westernization in humans and the hominids in general, and argue for the diverse interaction between host background, lifestyle, and microbiome as shown in previous studies on humans as well [31].

**Fig. 5:**
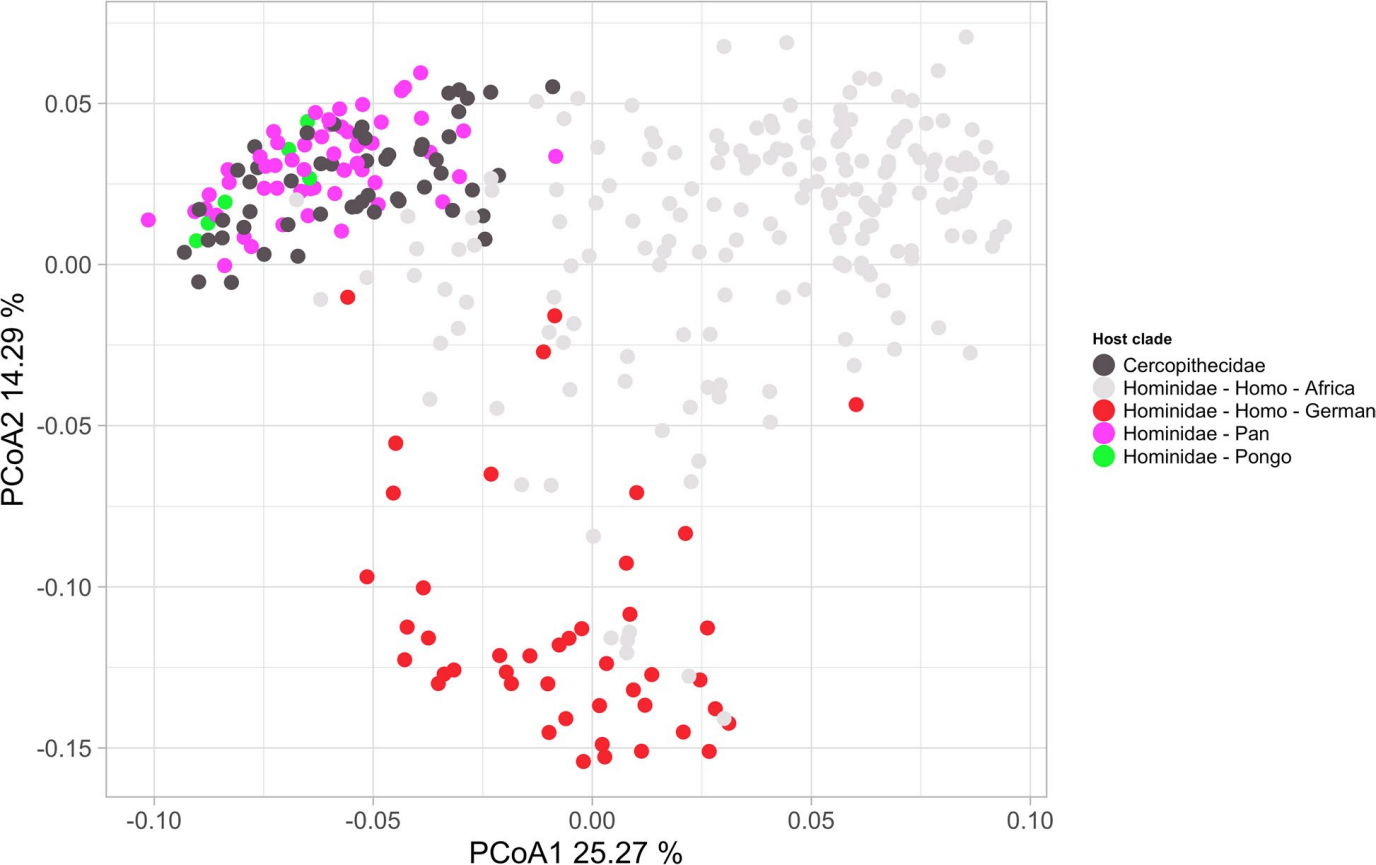
Community structure for humans and catarrhines . Samples ordinated by microbial Bray-Curtis dissimilarity showed a pattern of microbial composition dictated more by ecology than by host phylogeny, when comparing catarrhine monkeys to Hominidae individuals. The plot show host animals colored by phylogeny, and location for the humans. The first ordination axis separates the humans from the non-human species, while the second axis separates the westernized and rural human samples. The ordination is unconstrained principal coordinates analysis made with vegan::capscale, with dist = “bray”, metaMDS = F. Percentages given at each axis present the proportion of variance explained on the axis

There exist over 250 different species of monkeys, and these can be divided into two main groups; the catarrhines that are native to Africa and Asia, and the platyrrhines that are native to Central and South America. Our dataset also includes different species of platyrrhines (33) and catarrhine (52), with catarrhines falling in the *Cercopithecidae* family and platyrrhine in the *Callitrichidae* and *Cebidae* families. Recently, Amato and colleagues found evidence that human stool microbiota composition is more similar to members of the *Cercopithecidae* than to those of *Pan* and *Pongo* [22]. Thus, we here analyzed compositional differences using data from zoo animals instead of wild ones. Surprisingly, data obtained confirmed the overall findings of Amato et al., despite the close contact all animals in zoos have with humans. Comparisons of the microbiota composition (species) of the *Cercopithecidae* mammals with German humans, African humans and *Pan*, using adonis2, showed a smaller difference between German humans and *Cercopithecidae* (*R*^2^ = 0.36), as compared to German humans and *Pan* (*R*^2^ = 0.40) (Fig. 5). When taking the African humans instead of the German humans, the pattern was the same, however both measures of difference was reduced (vs. *Cercopithecidae R*^2^ = 0.21, vs. Pan *R*^2^ = 0.24).

## Discussion

Compared to the vast microbial diversity in most mammals, human gut microbiomes have lost diversity while becoming specialized for animal-based diets – especially compared to their genetically closest ancestors and particularly in westernized societies [13, 18, 32, 33]. Meanwhile numerous studies have shown that low diversity of the gut microbiome associates with different types of chronic diseases in humans [9, 10]. To further expand our understanding of the role of evolutionary history for shaping the gut microbiome and how this might impact today’s human health, we created this broad dataset and conducted comprehensive analyses.

In our dataset the highest taxonomic diversity was detected in the orders Artiodactyla and Perissodactyla. Though a high diversity within Artiodactyla and Perissodactyla has been shown in various studies before [12], we found high proportions of not assignable bacterial families highlighting a yet hidden microbial diversity within these host groups. In contrast to these hosts, we observed relatively less diverse but highly specific bacterial communities in samples from the order Carnivora, which are mainly composed of well characterized bacteria, as was also observed for the order Primates. In general, bacterial community structure largely followed the phylogenetic relationship of the studied mammals with a common core microbiome of at least 30 bacterial species (relative frequency normalized by group size above 50%) in all groups, except for Carnivora. This finding might reflect the functional assembly of bacteria shared between the omnivorous and herbivorous hosts among the Artiodactyla, Perissodactyla, and Primates as described previously [11, 34]. This core microbiota contains bacterial strains that are essential for the breakdown of plant fibers and production of SCFAs and are thus crucial for host physiology [35]. Due to the limitations of 16S rRNA gene amplicon sequencing we were not able to define specific strains and genes that form part of this core, however ongoing studies in our laboratory using deep shotgun sequencing will uncover those in the future and extend upon previous low coverage analyses.

As summarized by Groussin et al. in 2017 [34], the consistent dominant drivers of animal gut microbiome diversity appear to be host evolutionary history and diet (including physiological adaptations), while also biogeography, sex, reproductive status, and social structure have been associated to microbial community differences. While similar limitations apply to our dataset, captive animals’ dietary sources are more controlled and therefore easier to record. From an analytical point of view, dietary behavior of the here included animals largely follows their phylogeny, thus making the partitioning of microbial variation between diet and host phylogeny difficult. Dietary data was gathered for a subset of the dataset and allowed for an evaluation of the role of both diet and host phylogeny. Interestingly, by using the subset with dietary information our analysis suggests that the effect of host phylogeny is still stronger than that of diet. Previous studies focusing on the strictly herbivorous Panda bears underline this finding by demonstrating that their fecal gut microbiota is more similar to their carnivorous and omnivorous relatives than their dietary behavior suggests [33, 36]. It must be noted that the dataset comprises a limited number of carnivores (*n* = 6). Having samples from seven different locations available for analysis we could also demonstrate that host location only has a minor influence on the bacterial community structure of zoo animals. More precisely, location/geography appears to influence only individual bacterial families in most animals, whereas the broad taxonomic levels are dictated by host phylogeny as has been shown in previous studies [37–39]. It must be noted that samples from each zoo were shipped and processed in batches, which could contribute to the variation associated to location. We could not observe any bacterial clades that were location-specific and prevalent, most probably due to the central role of host phylogeny for bacterial prevalence patterns as well as the well-defined and shared diet and dietary information throughout German zoos, which may have a homogenizing effect. As most samples from zoos were collected collectively from zoo compounds, it was not feasible to include information on sex or reproductive status of the animals, which limits the interpretation of our study results regarding this known confounder in microbiome studies [40].

During the last years, the ecology of mammals has come into focus in microbiome research and surprisingly shows many parallels to patterns of human associated microbial communities and has been shown to even overcome host phylogeny [41–43]. In line with the study of Amato et al. 2019, we found that the fecal bacterial community between humans and individuals belonging to the platyrrhine monkeys is more similar than between humans and *Pan* or *Pongo*, which are phylogenetically closer [13, 23, 44]. As hypothesized by Amato et al., this effect might originate from the human dietary niche and associated physiological adaptations, which are more similar to those of cercopithecines than to other apes [23]. Thus, the findings of Amato et al. could be confirmed even in captive animals living in close contact with humans – strongly supporting the influence of phylogeny, ecology, and associated physiological adaptations in shaping the microbial community of primates.

After having assessed the importance of host phylogeny, location, and diet in shaping the gut microbiome in the full dataset, we turned our focus to the family Hominidae to further understand the observed decrease of fecal bacterial diversity in westernized humans during the last 100 years. To further explore this trend, we included not only a human cohort from North Germany, but also a dataset of fecal microbiotas determined from communities from Western and Central Africa. When comparing the fecal bacterial diversity between all host groups of the Hominidae family, we justified previous observations about the westernized (local to Kiel, Germany) humans: this cohort had the lowest diversity as well as the highest specificity and dispersion (reviewed in [33]). In addition, we could confirm earlier findings of reduced relative abundance of *Prevotella* – probably replaced by increased relative abundances of *Bacteroides* – and an increase of several clades that are associated with carnivorous diet in other mammals, e.g.*, Enterobacteriaceae* [33, 43]. This trend was also reported in studies comparing urban and rural human cohorts only and hypothesized to be due to consumption of sugar, animal fat, and calorie-dense foods in industrialized countries [14, 15]. Additionally, we observed a decrease of Spirochaetes specifically for the German (westernized) human cohort, highlighting a trend of urbanization-associated loss within the human species that was described in other studies as well [14, 21]. This finding, however, could be expanded on with this dataset as we observe high proportions of Spirochaetes in fecal samples from nearly all other mammal orders except for Carnivora and - more noteworthy - primates belonging to the platyrrhines. It has been speculated that the presence of Spirochaetes and *Prevotella* relates to the high fiber intake from ingested plant polysaccharides to produce high levels of short-chain fatty acids and thereby maximizing metabolic energy extraction [14, 21]. However, a study in baboons demonstrated that social interactions are an important determinant of gut microbiota composition as well [41]. Here, Tung et al., studied baboons belonging to two different social groups and found that direct physical contact during social interactions plays a role in transmitting gut bacteria between members of the same social group. Particularly, anaerobic and non-spore-forming bacteria such as Spirochaetes and *Prevotella* were found amongst those “socially structured” microbes [41]. This would indicate that the observed changes in abundances of Spirochaetes and *Prevotella* might also depend on host’s social behaviour. Though most platyrrhines are often described to live in monogamous pair bonds and live only in small groups [45], more recent literature show large amounts of variability in social organization in nature [46]. However, particularly in zoos, platyrrhines are often kept in pairs with limited contact to other species. Urban human communities also tend to live separated in small groups or families, whereas rural communities are living closely together, sharing smaller rooms and often live in close connection to their livestock. Thus, consistent changes in fecal microbiota composition among phylogenetically very close related mammals highly correlate with their social relationships. The samples from two zookeepers from Gettorf that cluster with the animals they are taking care of highly underlines this assumption though with very limited sample size in our study. However, the same observation has been made before for human family members that share the fecal microbiota with their dogs despite having different diets [47]. However, it shall be noted that Spirochaetes are mainly occuring in anaerobic sediments [48] to which both – urban human communities (mainly living in cities) as well as platyrrhines (mainly arboreal) – have less contact. These ecological characteristics could of course play an essential role for the loss of these bacteria and thus warrants future studies.

One of the main technical artifacts in studies elucidating mammal fecal microbiota has been noted to be sampling in zoos instead of wild animals [3, 12, 22, 25]. While confinement undoubtedly affect the animals at different levels, especially the overall microbial diversity in stool as has been described in various studies [49–53], we think that the herein studied mammals from different German zoos were more comparable when studying host phylogeny effects due to standardized food and rhythms in their diet. This is underlined by the limited microbial variation found to be explained by location or diet. In addition, the clustering of primate species highly reflects their phylogeny, though many of them live in different group sizes and together with a diverse range of species within the zoo itself. Recently, Nishida et al. also detected no significant microbial variation between wild and captive animals [24], though of course antibiotic influences have been reported [22] and other studies highlighted large transitions after the transfer of wild animals into laboratory environments [20]. Moreover, we could confirm various earlier observations that were made for the microbiome of wild animals. Thus, we find that it is of much higher importance to employ the same standardized and well-controlled sample processing pipeline throughout a study and well controlled environment to reduce potential confounders and to deduce the phylogenetic patterns inherent to the microbiome composition. In addition, while effects of location and housing conditions are present and an important factor in microbiome studies, the role of host phylogeny remains strong and appears resilient to several confounders.

## Conclusion

In conclusion, our study highlights the importance of phylogenetic relationship and ecology within the evolution of mammals’ fecal microbiota composition. Particularly, the notable decrease of Spirochaetes and *Prevotella* in westernized communities might be associated to lifestyle dependent evolutionary fast-track changes, potentially involved in the establishment of less diverse microbiomes with association to the etiology of chronic diseases [54]. Consequently, the observed findings urgently need deeper analysis based on shotgun metagenomics and metatranscriptomic studies to gain insights into the functional loss as well as the immunogenic consequences that might be associated.

## Materials and methods

The STORMS (Strengthening The Organization and Reporting of Microbiome Studies) Checklist has been used to check all relevant information is provided for the study, and the checklist can be found in Table S5.

### Cohort sampling

For this cohort study, stool samples from animals were collected between 2015 and 2017 in seven different, randomly selected zoos within Germany (Arche Warder, Gettorf and Neumuenster from Schleswig-Holstein, Berlin, Hagenbeck in Hamburg, Leipzig in Sachsen and Schwaigern in Baden-Württemberg) by the responsible zookeepers and kept frozen at − 80 °C until shipment to the laboratory. Zookeepers ensured sample origin, excluding animals given antibiotics in past 6 months and provided dietary and relationship information on the animals included in this study. The dietary data was thereby provided by different individuals with the inherent risk of bias. Samples were otherwise treated the same and processed in the same lab.

To be able to find potential relationships between dietary factors, phylogenetics as well as dysbiosis associated to the western-lifestyle, stool microbiome samples from 44 humans were included in the analysis (in addition to four zookeepers from Gettorf). The 44 subjects were selected as aged 30-40, normal weight and no disease reported, from a cohort recruited at the University Hospital Schleswig Holstein, Campus Kiel 2016 and comprised detailed phenotypic, disease related and dietary information. The study was approved by the local ethic committee in Kiel (D441). None of the participants had received any antibiotics or other medication 2 months prior to inclusion, and none reported any gastrointestinal complaints.

### Stool sample processing and sequencing

Samples from zoos was shipped in batches and processed in these batches. DNA of feces samples was extracted using the QIAamp DNA fast stool mini kit automated on the QIAcube (Qiagen, Hilden, Germany). Therefore, material was transferred to 0.70 mm Garnet Bead tubes (Dianova, Hamburg, Germany) filled with 1.1 ml InhibitEx lysis buffer. Bead beating was performed using a SpeedMill PLUS (Analytik Jena, Jena, Germany) for 45 s at 50 Hz. Samples were then heated to 95 °C for 5 min with subsequent continuation of the manufacturer’s protocol. Extracted DNA was stored at − 20 °C prior to PCR amplification. Blank extraction controls were included during extraction of samples.

For sequencing, variable regions V1 and V2 of the 16S rRNA gene within the DNA samples were amplified using the primer pair 27F-338R in a dual-barcoding approach according to Caporaso et al. [55] Stool DNA was diluted 1:10 prior PCR, and 3 μl of this dilution were finally used for amplification. PCR-products were verified using the electrophoresis in agarose gel. PCR products were normalized using the SequalPrep Normalization Plate Kit (Thermo Fischer Scientific, Waltham, MA, USA), pooled equimolarily and sequenced on the Illumina MiSeq v3 2x300bp (Illumina Inc., San Diego, CA, USA). Negative controls, tubes with reagents from DNA extraction and water only, and positive controls, known human stool sample, was included. Demultiplexing after sequencing was based on 0 mismatches in the barcode sequences. All laboratory work was conducted in the microbiome lab of the IKMB at Kiel University, Germany, except DNA extraction of the adult African samples as was performed at RKI in Berlin.

### Data processing

Data processing was performed using the DADA2 [56] workflow for big datasets (https://benjjneb.github.io/dada2/bigdata.html) resulting in abundance tables of amplicon sequence variants (ASVs) with mean 26,676 reads/sample and min 5995 reads for the zoo dataset. Briefly, all sequencing runs were handled separately (workflow adjusted for V1-V2 region can be found here: https://github.com/mruehlemann/ikmb_amplicon_processing/blob/master/dada2_16S_workflow.R) and finally collected in a single abundance table per dataset, which underwent chimera filtering. ASVs underwent taxonomic annotation using the Bayesian classifier provided in DADA2 and using the Ribosomal Database Project (RDP) version 16 release. Sequences that were not assignable to genus level were binned into the finest-possible taxonomic classification. Prior to further analysis, two samples were removed as they came from animals with only one representative species per family, and seven samples were removed as they belonged to the outlying sparsely represented order Diprotodonts, which incorporates marsupial mammals only and whose occurrence is restricted to Australasia. ASV abundance tables and taxonomic annotation were passed on to the phyloseq package for random subsampling to 5900 sequences per sample (rarefy_even_depth(), removing 6998 OTUs) or calculation of relative abundance (normalized by rowsum, minimum read depth 5900) and construction of phylum- to species-level abundance tables (tax_glom(x, NArm = F)).

The final dataset comprised 368 samples, from 38 unique hosts, unknown host or “collective faecal samples”, including 48 humans. A number of samples were collected from “collective faecal samples” in cages and the specific host species animal is therefore not known. For the remaining samples, the specific host animal was recorded by name or no specification was given. In analysis that include one sample per host, we removed all samples but one from named animals, and kept samples from unnamed or “collective faecal samples”.

To include a profile of non-westernized human gut microbiome, two additional datasets were included. One dataset was generated from a large cohort of 1204 subjects with fecal bacterial microbiota data, collected in one of the poorest countries in the world, Guinea-Bissau, Western Africa, between 2015 and 2017, as described previously [27]. Data was processed with DADA2 and turned into a phyloseq object, after which a subset of 159 subjects were selected as the subset recruited at home (controls), with no recent antibiotic usage and min. 10 years old. The second dataset comprised gut microbiome data from 24 adults, 12 from Ivory Coast and 12 from DR Congo. The dataset was presented in Gogarten et al. 2021 [28]. After DADA2 processing and conversion to phyloseq objects, both datasets were joined with the data described above to evaluate the associations of present lifestyle vs phylogeny.

### Data analysis

A phylogenetic “timetree” was built for mammal species using the website http://timetree.org/ and the resulting tree was imported to R as a phylo object and used throughout the analysis by sub-setting to species of interest for each specific analysis. A list of 42 mammal species was uploaded.

To assess tree topology congruence, two measures were analyzed: The phytools::multiRF function was used to calculate the Robinson-Foulds distance between the host phylogenetic tree and the microbial dendrogram (Bray-Curtis and Jaccard). The analyses was repeated 100 times, each time randomly selecting one sample to represent each host-species group, and to estimate significance, a null-distribution was generated by shuffling (base:: sample) tip labels of the host tree 1000 times. The vegan::mantel analysis was used to compare diversity matrices based on host phylogenetic (applying stats::cophenetic to the phylogenetic tree) and the microbiome beta-diversity matrices, again repeated 100 times for randomly selected host-species representatives.

Specificity of individual microbial taxa to subgroups of hosts was evaluated using the ﻿indicspecies::multipatt function in R with settings func = “IndVal.g” and control = how(nperm = 10,000) for analysis within Hominidae and control = how(blocks = (factor(location)), nperm = 10,000) for analysis across host order clades. The function calculates if a taxon show specificity to any one or a combination of subgroups as defined by a cluster argument. For the identification of microbial indicator species for mammal order clade(s) or human subgroup (Kiel-area or African), species were first filtered to keep those present in at least 0.5% of samples, and only mammal subgroups with min. Five subjects were included in the analysis. Tables of species abundance and prevalence across host groups was also generated using the multipatt function and used for visualization in Fig. 4.

Alpha diversity was calculated from rarefied count data using diversity() and estimateR() from vegan R package. Differences in alpha diversity between groups was analyzed using ANOVA (stats::aov) while correcting for zoo location. To evaluate the number of indicator species specifically for a host subgroup, e.g. host order groups, the data was prepares as for multipatt, and analyzed with indicspecies::indicators with settings At = 0.5, Bt = 0.10, max.order = 1, func = “IndVal.g”, control = how(blocks = factor(location), nperm = 10,000).

For dispersion analyses of mammals grouped by dietary behaviour (Carnivore, Herbivore, Omnivore) we performed dispersion analysis using vegan::betadisper with bias.adjust = T. To evaluate if the results were robust to variation in the number of different host species clades found in each diet group, we performed random selection of five host species for the two diet-groups herbivores and omnivores and performed the dispersion analysis. We did so 100 times and calculated the mean dispersion and *p*-value using ANOVA. The Carnivora were not included due to very few different species in this group. The analysis was performed for microbial taxa at the ASV, species, genus and family level, filtered to keep taxa present in at least 1% of samples. For dispersion analyses including the African samples, species level data was used and the filter threshold set to 0.5%, and significance evaluated using ANOVA directly on betadisper objects. Permutational MANOVA analysis was performed using vegan::adonis2, with species level data filtered as for betadisper. Animals clades were included if they contained min five samples, and analysis were run with 999 permutations and method = “bray”. Analysis of phylogeny was correcting for zoo, and vice versa.

The multiple regression on matrices (MRMs), as described previously [57], was used to calculate how much of the microbial variation could be assigned to host phylogeny and location (ecodist::MRM with 1000 permutations). For each MRM analysis, one sample was selected per host species, and this was repeated 100 times. Then the median coefficient and *p*-value was calculated. The three-distance matrix was based on geographic coordinates for location (distGeo() function in geosphere), patristic distances for host phylogeny obtained from the website http://timetree.org/ (see above) and the species table for the microbiota filtered to keep species present in > 1% of host animals (602 species). Bray Curtis dissimilarity and Jaccard distance was calculated using vegan::vegdist() function in R. Details on diet was available for 115 hosts comprising 15 species. The data was used to generate eight binary dietary variables that reflect the main dietary categories that could be extracted from the available information on the animal’s diets. The eight binary variables included in the analysis (fruit, meat, vegetables, eggs, greenery, Herbs/tea, multivitamin/mineral and vitamin B) were used to generate a distance matrix based on the binary Jaccard distance. For the PGLS analysis the species microbiome profiles were used, with the host phylogenetic timetree expanded to include all samples as for MRM, and analysis performed using the caper:: comparative.data and caper::pgls functions with lambda = ‘ML’. For the MPD analysis, data was prepared as for PGLS, but with the extra step of regressing out the effect of location using a linear regression with square root transformation of taxa abundance and selecting species present in > 5 samples. The MPD analysis was performed given the microbiome species community matrix and a distance matrix for the hosts calculated from the host phylogeny using stats:: cophenetic, to the function picante::ses.mpd, with the settings null.model = “richness”, abundance.weighted = T and runs = 999.

Then, we looked for zoo-specific microbial species. The microbiome species were filtered to keep the 348 most abundant microbial species (min abundance 1e-5 in at least 3% of the 368 samples), and data converted to presence/absence. Using this data, we identified species only present in one location.

### Illustrations

To generate stacked barplots, taxa with relative abundance not more than 0.05 in at least 10% of the samples were removed, remaining taxa renormalized to sum to 100 per sample, and plots made using function barplot in R package ﻿graphics. The plot.phylo() function in R package ape v5.3 was used to generate the phylogenetic tree in Fig. 1. Boxplots were generated using ggplot. Figure panels were arranged using ﻿function ggpubr::ggarrange, or cowplot::plot_grid, and colors selected from RColorBrewer. Ordination plots in Fig. 2 were made using vegan:: capscale with microbiome data aggregated at species level and filtered to species present in at least 1% of samples, dist = bray and metaMDS=F, and ggplot for illustration, while microbial species for Fig. 5 were filtered to species present in at least 0.5% of samples. The heatmaps in Fig. 4 were made using data extracted from the multipatt analysis, and pheatmap:: pheatmap.

## Supplementary Information

**Additional file 1: Table S1.** Species association with host order clades. **Table S2.** Phylogenetic relatedness of microbial species. **Table S3.** Microbial species community association with host phylogenetic groups and location. **Table S4.** Species association with host subgroups in the Hominidae family. **Figure S1.** Microbial alpha diversity along host family clades and location. **Figure S2.** Microbial Chao alpha diversity along host order clades and location. **Figure S3.** Gut microbiota community variation mainly explained by mammal phylogeny as opposed to location. **Figure S4.** Microbial Shannon (A) and Chao (B) alpha diversity along hosts grouped by genus with humans further segregated by location and for Africans, adult versus children cohort.

**Additional file 2: Table S5.** STORMS Checklist.

## Data Availability

The datasets generated and analysed during the current study are available at the NCBI SRA repository (Accession no. PRJNA685981 and PRJNA642721). Codes used for data analyses and supportive data files are available at https://github.com/LouiseBThingholm/Zoo-Microbiome-Project-2020. We would like to emphasize that all stool samples, as well as the corresponding extracted DNAs are available at the Institutes biobank for non-profit research purposes upon request.
