# Supplementary material for: Ecology impacts the decrease of Spirochaetes and Prevotella in the fecal gut microbiota of urban humans
Source: BMC Microbiol. 2021 Oct 11;21:276. doi: 10.1186/s12866-021-02337-5 (PMC8504008; doi:10.1186/s12866-021-02337-5)
Supplement: Supplementary file 1 — Additional file 1: Table S1. Species association with host order clades. Table S2. Phylogenetic relatedness of microbial species. Table S3. Microbial species community association with host phylogenetic groups and location. Table S4. Species association with host subgroups in the Hominidae family. Figure S1. Microbial alpha diversity along host family clades and location. Figure S2. Microbial Chao alpha diversity along host order clades and location. Figure S3. Gut microbiota community variation mainly explained by mammal phylogeny as opposed to location. Figure S4. Microbial Shannon (A) and Chao (B) alpha diversity along hosts grouped by genus with humans further segregated by location and for Africans, adult versus children cohort. [file 12866_2021_2337_MOESM1_ESM.zip › BMC_microbiology_Suppl_revision.docx]

# Supplementary Information

**Table S1: Species association with host order clades.** A ﻿multi-level pattern analysis was used to evaluate the association of gut microbial species with each of the four host order clades in the dataset (multipatt in R package ﻿indicspecies, func = " IndVal.g", control = how(nperm=999), regress out effect of location on species). The analysis identified 305 species with significant specificity (out of 602 tested) to any one combination of host order clades (p.adj<0.05). The table shows associations to one or more host order clades for every tested microbial species, along with statistics from multipatt analysis and species annotation.

**Table S2: Phylogenetic relatedness of microbial species.** Analysis of phylogenetic relatedness for 536 species identified 231 significant species (picante::ses.mpd, abundance weighted MPD model, 999 permutations, p.adj<0.05). The table show statistics from the ses.mpd analysis for each species (rows) and the species annotation. The statistics columns; ntaxa: Number of taxa in community, mpd.obs: Observed mpd in community, mpd.rand.mean: Mean mpd in null communities, mpd.rand.sd: Standard deviation of mpd in null communities, mpd.obs.rank: Rank of observed mpd vs. null communities, mpd.obs.z: Standardized effect size of mpd vs. null communities (= (mpd.obs - mpd.rand.mean) / mpd.rand.sd, equivalent to -NRI), mpd.obs.p: p-value (quantile) of observed mpd vs. null communities (= mpd.obs.rank / runs + 1), mpd.obs.p.adj: multiple testing adjusted p-value.

**Table S3: Microbial species community association with host phylogenetic groups and location.** ﻿Permutational MANOVA (vegan::adonis) was used to evaluate the association between gut microbiome composition and host clades and location (species-level microbiome, 999 permutations, min. 5 animals per host clade). The analysis was performed for hosts grouped at order to species levels (rows) and adjusting for location when analyzing phylogeny, and visa versa. All analyses supported a significant and dominating role of host phylogeny in shaping the gut microbiome, but also highlighted a role of location for shaping the microbial composition.

**Table S4: Species association with host subgroups in the Hominidae family.** A ﻿multi-level pattern analysis was used to evaluate the association of gut microbial species with each of the four host groups; humans from Africa or Germany (Kiel-area), *Pan* and *Pongo* (multipatt in R package ﻿indicspecies, func = "IndVal.g", control = how(nperm=999)) Effect of location was not regressed out due to the unvaried sampling of humans in Germany. The analysis found 65 species (out of 351 analyzed) with significant specificity to one host group, with 2 assigned to Guinea-Bissau humans, 36 to German humans, 8 to Pan and 19 to Pongo (multipatt p.adj<0.05). The table shows associations to one or more host order clades for every tested microbial species, along with statistics from multipatt analysis and species annotation. NA is listed for species whose highest IndVal-value correspond to all host groups.

**
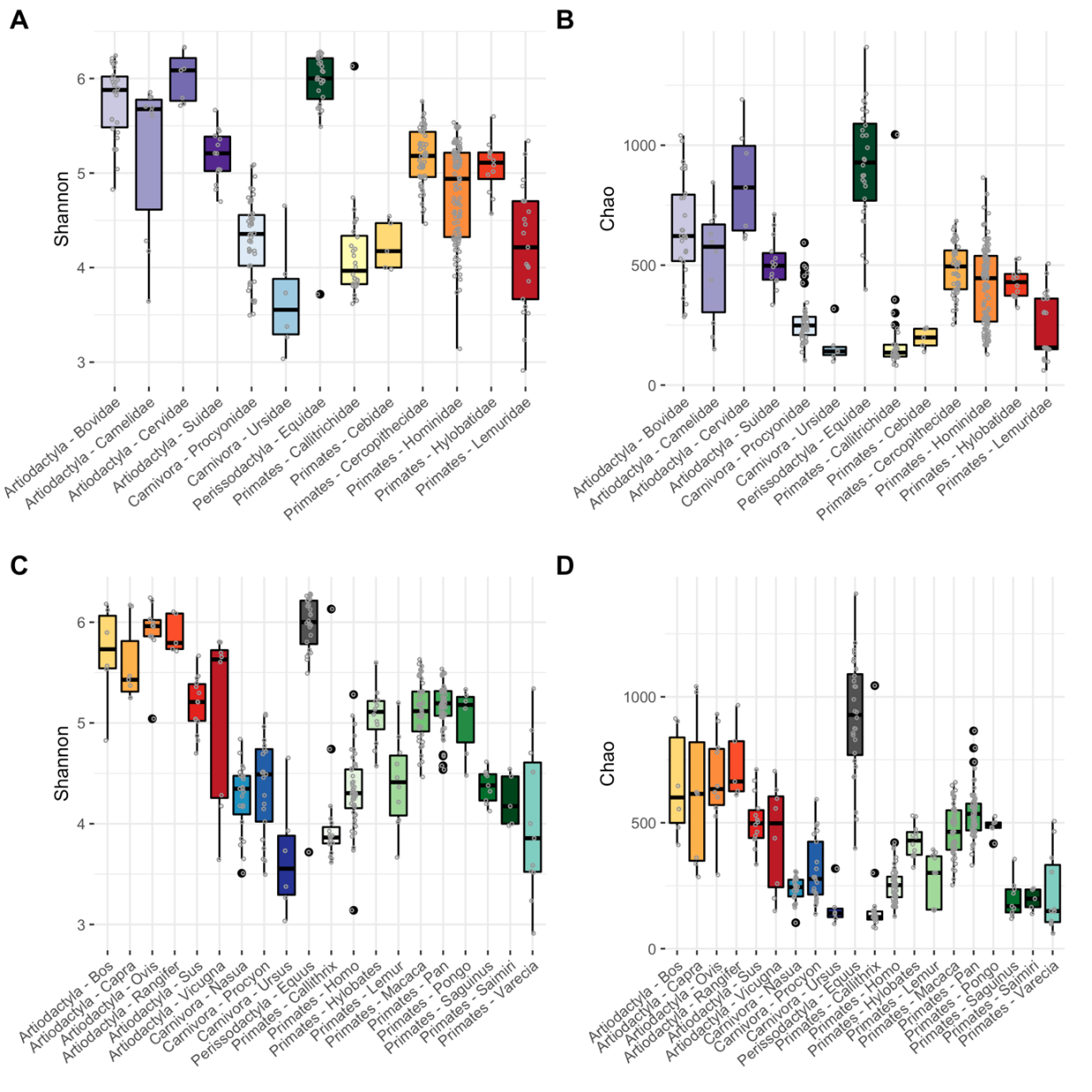
**

**Figure S1: Microbial alpha diversity along host family clades and location.** Comparison of the alpha diversity measures Shannon (A, C) and Chao (B, D) between host clades (A-B) at family-level and (C-D) genus-level.


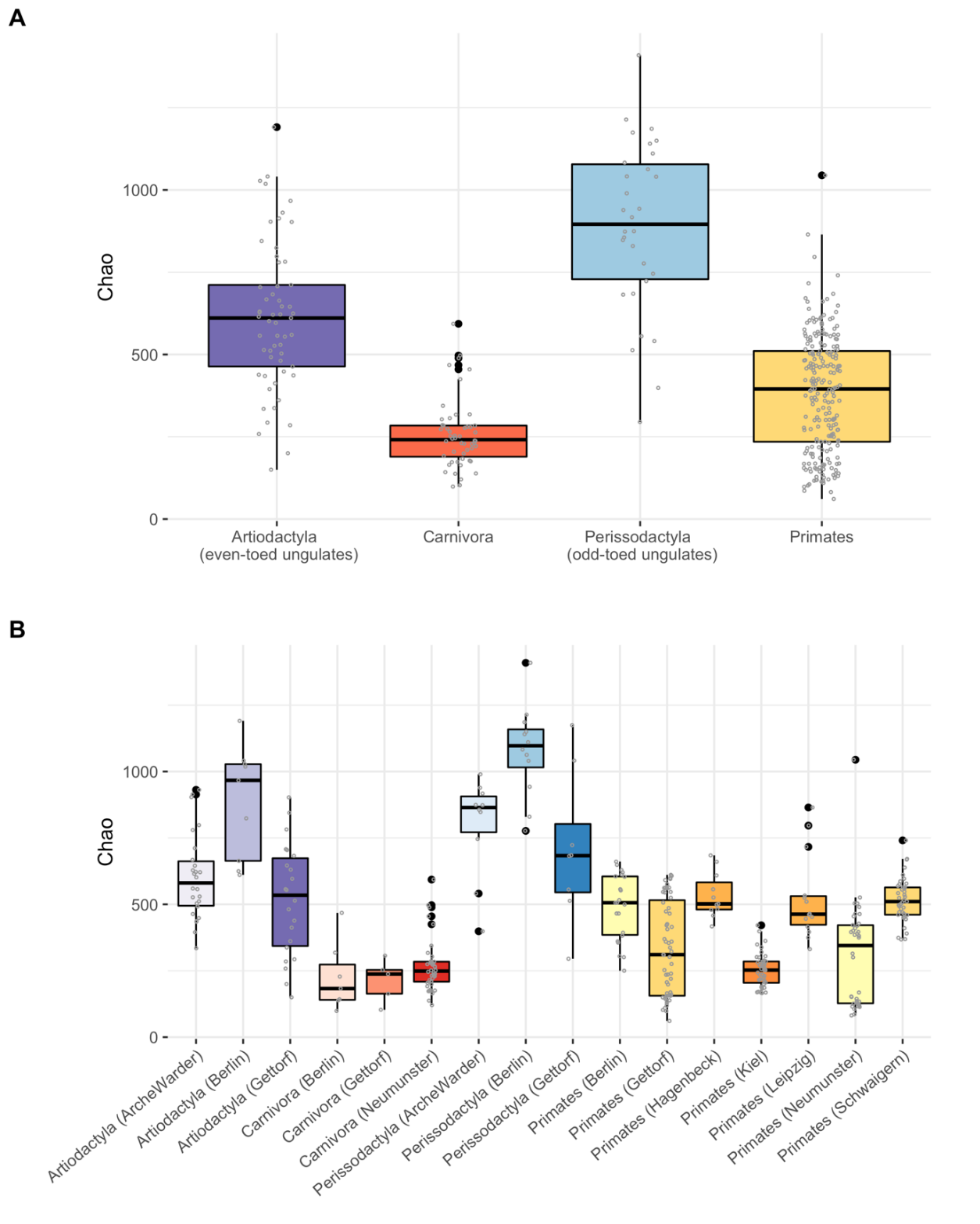


**Figure S2**: **Microbial Chao alpha diversity along host order clades and location.** Comparison of the alpha diversity measure Chao between host clades (A) at order-level and (B) sub-stratified by sampling location.

| **Bray-Curtis** | | **Jaccard** | |
| --- | --- | --- | --- |
| 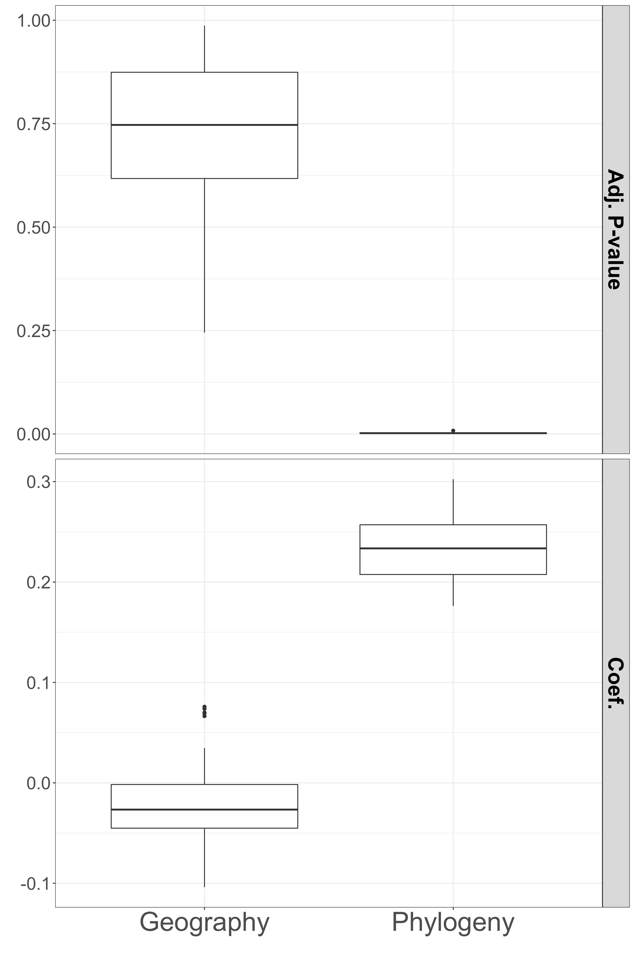 | | 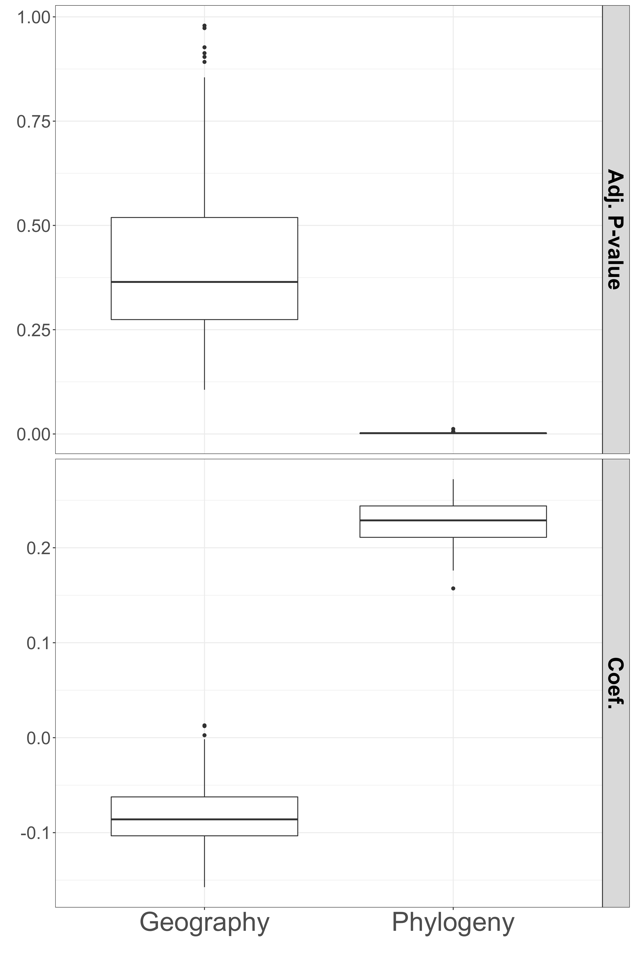 | |
|  |  |  |  |

**Figure S3**: **Gut microbiota community variation mainly explained by mammal phylogeny as opposed to location.** Multiple regression on matrices (MRMs) was used to evaluate the gut microbiome (species) variation that associated with phylogeny and location, with location given by the Zoo’s geographical locations and humans home-country. The analysis was performed for both relative abundances (**left**) and presence/absence (**right**) of the microbiota species composition. In both analyses, host phylogeny explained a significant amount of variation (median p-value<0.05), while the variation explained by location was insignificant (median p-value>0.05).

**
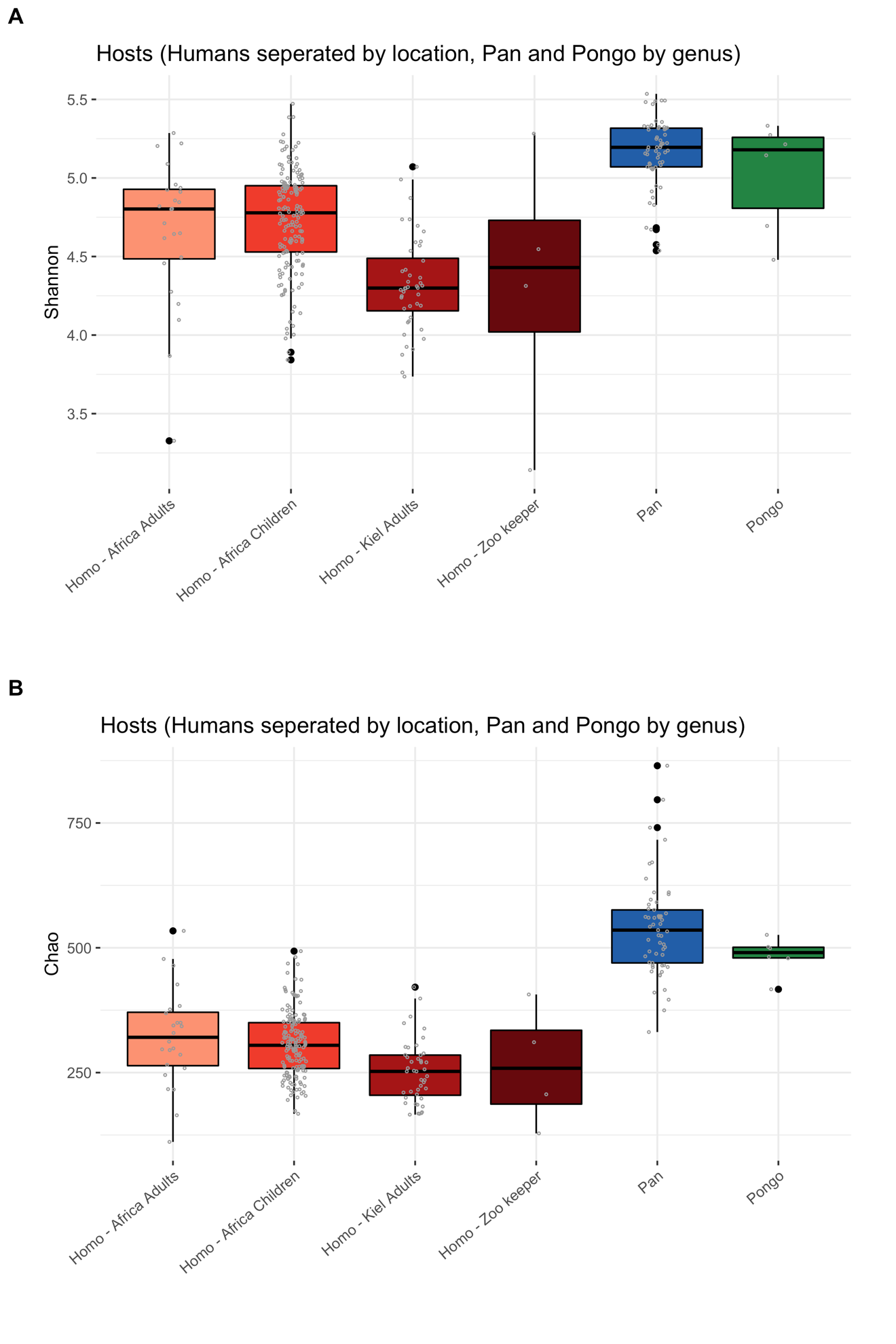
**

**Figure S4**: **Microbial Shannon (A) and Chao (B) alpha diversity along hosts grouped by genus with humans further segregated by location and for Africans, adult versus children cohort.** Comparison of the alpha diversity measure Shannon between host clades and location for humans, showed a higher diversity for the none-human mammals, followed by African human subjects and then German human subjects. A small subgroup of zookeepers (n=4) showed a large spread in diversity.
